# Developing Customized Personas to Capture Intrinsic Capacity Profiles and Digital Monitoring Intentions in Older Adults: Mixed Methods Study

**DOI:** 10.2196/82867

**Published:** 2026-05-27

**Authors:** Zehui Xuan, Yirou Niu, Ruifu Kang, Qian Xiao, Shuai Jin, Jie Zhao, Yanling Wang, Hong Chang

**Affiliations:** 1 School of Nursing Capital Medical University Beijing China; 2 Department of Neurology Xuanwu Hospital, Capital Medical University Beijing China

**Keywords:** digital monitoring, integrated care for older people, intrinsic capacity, latent profile analysis, mixed methods study, older adults, personas

## Abstract

**Background:**

Integrated Care for Older People (ICOPE), focused on monitoring and optimizing the intrinsic capacity (IC) of older adults, is a new model of geriatric care that is currently being accelerated globally. Digital health technologies are recommended for longitudinal IC monitoring to provide precise and timely interventions. However, little is known about the psychological intentions of engaging in digital monitoring of IC according to the profile heterogeneity of IC among older adults.

**Objective:**

This study aims to map a set of customized personas to capture the profiles of IC and match psychological intentions that support personalized digital IC monitoring.

**Methods:**

An explanatory sequential mixed methods study was conducted at 16 sites in Beijing, China. Older adults aged ≥60 years (n=481) were selected to complete the quantitative survey. Latent profile analysis, descriptive statistics, and logistic regression analyses were performed to cluster subgroups using Mplus (Muthén & Muthén) and SPSS (IBM Corp). A subsample of participants from each profile (n=25) was purposively sampled for semistructured interviews. An inductive-deductive content analysis was used to identify similar attributes and to affirm the personas gradually. A joint statistical and thematic visualization method was used to integrate the customized personas.

**Results:**

Three profiles of IC patterns emerged: “multisubdomain decline–IC imbalance group,” “multisubdomain moderate–sensory deficit group,” and “multisubdomain robust–whole balance group.” The distribution of latent profiles was influenced by age, education, monthly per capita household income, self-rated health, and number of chronic diseases, while positively impacting older adults’ functional ability. The following customized personas were captured regarding established themes: “affects my mood—anxious evader,” characterized by avoidance and anxiety, low digital interest, and perceived social isolation; “capitalize on what comes—accommodative adopter,” pragmatically oriented toward disease detection, with moderate digital openness but limited self-efficacy; and “more autonomy—active improver,” who exhibited proactive engagement, high digital literacy, and motivation rooted in self-management and social participation.

**Conclusions:**

This study is the first to integrate latent profile analysis with customized qualitative personas to link the heterogeneity of IC with the psychological intentions underlying digital monitoring. The resulting personas model provides an actionable framework for tailoring digital IC monitoring strategies in community-based integrated care. The findings emphasize the need to align monitoring approaches with older adults’ IC characteristics, psychological readiness, digital literacy, and social support to enhance engagement in digital IC monitoring.

## Introduction

### The Aging Challenge and the Centrality of Intrinsic Capacity

In the face of increasing aging trends, health care systems are confronted with a diverse and intricate array of geriatric care demands [1]. The transition from a model of health care that is centered on responding to diseases to one that focuses on maintaining functions is an urgent strategy for improving and coordinating health care services [2]. Intrinsic capacity (IC), commonly consisting domains of cognition, psychological capacity, sensory capacity (including vision and hearing), vitality, and locomotor capacity, is the physiological reserve of an individual’s functional ability and plays an important role in quality of later life [3,4]. It is defined as “the composite of all the physical and mental capacities that an individual can draw on” [1]. To operationalize this concept, the World Health Organization (WHO) has released 2 editions of the “Integrated Care for Older People (ICOPE): Guidance for Person-centered Assessment and Pathways in Primary Care” handbook to guide the assessment and monitoring of older adults’ IC in community settings [1,5].

IC, as a health metric reflecting the degree of healthy aging in a country or region, is of special reference value [6]. A systematic review stated that the global prevalence of IC decline among community-dwelling older adults was 67.8% [7]. In China, Ma et al [8] reported that 69.1% of participants showed a decline in 1 or more domains of IC. A longitudinal study of retired Chinese older adults revealed that 32.1% experienced a decline in IC over a 4-year follow-up period [9]. According to previous studies, IC is a robust predictor of adverse health outcomes, including frailty, falls, health care use, incident disability, hospitalization, and mortality [10,11]. In general, optimizing IC in older adults can enhance functional autonomy and predict adverse events. Consequently, dynamic monitoring of cognitive impairment is needed to identify risk factors that can be ameliorated and to develop individualized intervention strategies that delay or prevent the deterioration of IC.

### Digital Health for IC Monitoring: Potential and the Challenge of Equity

Digital health technology is an efficient and manageable approach for expanding the study and monitoring of IC in older adults [12]. An ICOPE program in France used digital tools to establish a timely feedback mechanism and support network for early warning IC monitoring in community settings, enabling interaction between older adults and health care professionals and providing an interface for subsequent steps in ICOPE [13-15]. A qualitative study indicated that implementing eHealth interventions within the IC framework may promote the social participation of older adults and communication with physicians, enhancing subjective well-being and enriching the monitoring activities of medical staff [16]. Currently underway in China, the “Comprehensive Evaluation of Intrinsic Capacity in China Study” (HEALTHY) aims to develop a consolidated digital technology–based platform for assessing IC and tailoring personalized interventions to promote proactive health behaviors among Chinese older adults [12]. However, older adults’ motivations for participating in digital health programs are complex and influenced by multiple factors [17]. Several studies have shown that older adults who engage in IC-focused digital health programs tend to be younger and healthier [13,14], which appears to conflict with the health equity goals advocated by ICOPE. The psychological intentions of older adults have a profound effect on their interaction with technology and their ability to use digital tools [18]. Therefore, establishing evidence-based links between IC heterogeneity and digital engagement patterns is an important process for scaling up the individual-centered ICOPE model and promoting equity in digital health for older adults.

### A Persona-Based Design for Customized IC Monitoring Strategies

The concepts of people-oriented integrated care and user-centered digital design achieve similar purposes and effects, both of which aim to meet the specific needs and preferences of the target group and achieve a deep and intuitive understanding [19]. Personas are a valuable approach for capturing older adults’ preferences, abilities, and attitudes toward self-management of health, particularly in the use of digital technology [20]. The novel paradigm of IC as a health criterion undoubtedly impacts the traditional disease-centered perceptions of older adults. Previous studies exploring the heterogeneity of IC subgroups have neglected to provide insight into older adults’ psychological intentions regarding IC monitoring and management [21,22]. Strategies for adopting digital health technologies may be hindered by a lack of demand or skills if only the external characteristics of older adults are targeted and the underlying behavioral determinants of accepting IC monitoring are overlooked [23]. Customized personas, with the advantage of incorporating biopsychosocial characteristics that encompass needs, experiences, and expectations, are an important reference for clustering older adults for digital IC monitoring [24].

### Study Purpose

Few studies have combined quantitative analysis with additional data to construct accurate customized personas, which are usually generated through gradual affinity grouping after labeling qualitative data [25,26]. To the best of the authors’ knowledge, this study is the first to adopt a mixed methods approach aimed at exploring the psychological intentions of older adults to receive integrated care focused on digital IC monitoring, thereby supporting personalized monitoring activities for community health care providers.

## Methods

### Study Procedures

**Figure 1:**
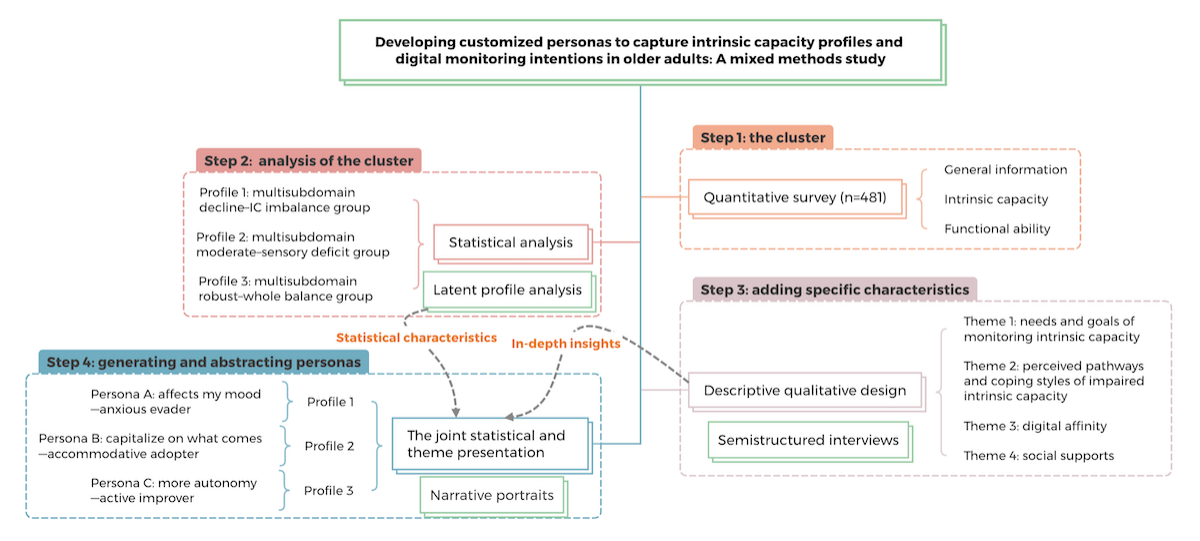
Study steps and integration process.

This study used an explanatory sequential mixed methods design, which was implemented in 2 consecutive phases [27]. First, a quantitative phase was conducted using structured questionnaires to collect objective data and identify key patterns or clinically significant issues. Subsequently, a qualitative phase followed, in which in-depth interviews were carried out with a purposively selected subgroup of participants to explore and interpret the underlying reasons and contextual meanings behind the quantitative findings [28]. The following hypotheses were proposed in this study: (1) by using the 5 domains of IC as observational indicators, older adults will be clustered into distinct groups, reflecting differences in IC levels and subdomain characteristics; and (2) older adults in differently characterized IC subgroups will express different motivations, goals, and concerns regarding digital monitoring of IC. A total of 3 PhD candidates in nursing jointly developed the research protocol under the guidance of 2 nursing professors, 1 associate professor, 1 lecturer, and 1 senior nurse practitioner, all of whom possesses extensive research or clinical practice experience in geriatric or community nursing. Figure 1 illustrates the 4 steps of the study [29], including: (1) the cluster: questionnaire survey on IC of older adults; (2) analysis of the cluster: to gain an initial understanding of differential characteristics and classification of subgroups based on latent profile analysis (LPA); (3) qualitative interviews to explore the attitudes, goals, needs, and potential concerns of older adults for digital monitoring under the different profiles of IC; and (4) integration and corroboration of quantitative and qualitative findings to portray a set of personas of older adults’ IC profiles and matched digital monitoring intentions, laying the groundwork for the future development of personalized monitoring strategies. Reporting adhered to the Good Reporting of a Mixed Methods Study (GRAMMS) checklist (Multimedia Appendix 1) [30].

### Quantitative Phase

#### Setting, Participants, and Recruitment

The study took place at a total of 16 sites, including 14 community committees, 1 nursing home, and 1 community health service center in 6 districts of Beijing, China, from March 2024 to October 2024. Convenience sampling was applied during the quantitative survey phase, as it aligns with the community-level screening and voluntary nature of IC assessment under the ICOPE framework. The authors coordinated and communicated with community workers and heads of health facilities before each questionnaire survey and recruited a consecutive sample of older adults by posting group notices and conducting onsite outreach after consent was obtained. Older adults who met the following inclusion criteria were eligible to participate in the survey: (1) aged 60 years or older; (2) with normal reading, writing, and comprehension skills; and (3) voluntarily participated in this survey after providing informed consent. The exclusion criteria were as follows: (1) participants with severe functional impairments that prevented them from safely completing an exercise test, (3) participants with medically diagnosed severe cognitive impairment, (3) participants with medically diagnosed serious psychiatric disorders, and (4) participants currently experiencing an acute or worsening illness.

The sample size was estimated based on 2 primary considerations. First, the initial target sample was calculated using the Kendall model, which recommended at least 5 to 10 times the number of variables [31]. With 17 variables in this study, and considering the possibility of 20% invalid questionnaires, the required sample size was 102 to 204 cases. Second, to ensure the stability and reliability of the subsequent LPA, a sample size exceeding the commonly recommended minimum of 300 is advised [32]. A final sample of 481 older adults therefore met both the adjusted recruitment target and the methodological requirement for robust LPA.

#### Variables and Instruments

Based on the literature review [33,34], a structured questionnaire was designed by the research team to collect general information as predictor variables, including sex, age, education level, marital status, family structure, type of labor force before retirement, per capita monthly family income, self-rated health using a Likert scale, and the number of chronic diseases.

The functional ability of participants, which results from the interplay between an individual’s IC, supportive environment, and personal characteristics [1], was assessed using 3 established scales as key outcome measures: the Barthel Index [35], Fried Frailty Phenotype [36], and HALFT scales [37] were used to assess activities of daily living, frailty, and social frailty, respectively.

The 5 key domains of IC were assessed following the instruments recommended in the new version of the WHO ICOPE guidelines [1]. The Mini-Mental State Examination [38], Geriatric Depression Scale-15 [39], WHO vision screening chart [1], whisper voice test [1], Mini Nutritional Assessment Short-Form [40], and Short Physical Performance Battery test [41] were used to assess cognition, psychological capacity, sensory capacity (vision and hearing), vitality, and locomotor capacity, respectively. The scoring rules of these instruments are summarized in Multimedia Appendix 2.

#### Data Collection

Before the formal questionnaire, the investigative team, comprising postgraduate nursing students, underwent a uniform offline training on the purpose of the study, assessment procedures, questionnaire content, assessment site setup, and language and behavioral norms. For participants who had difficulty completing the questionnaire, such as those experiencing presbyopia or slow reading speeds, the investigators helped them complete it after explaining the meaning of each item. The survey was conducted using either a paper version of the questionnaire or an application installed on tablet computers, with the content being the same in both formats. The paper version of the questionnaire was entered into the application after validity was established so that the data could be accessed for statistical analysis. At the end of the questionnaire survey, the authors invited older adults who were interested in this study to participate in follow-up qualitative interviews and retained their contact details.

#### Data Analysis

The IC of older adults is influenced by many factors and exhibits differential characteristics [42]. Therefore, we attempted to find a people-oriented clustering approach to provide an objective population classification basis for customized personas [32]. LPA is a statistical method that identifies characteristics between individuals based on the estimation of their response patterns on observed indicators, providing unique insights into the heterogeneity of the target population [43].

First, Mplus (version 8.3, Muthén & Muthén) was used to analyze the latent profiles of the IC of older adults. Starting with 1 category, categories were added progressively until the maximum likelihood method was used to determine the best solution. The main evaluation indices included Akaike information criterion (AIC), Bayesian information criterion (BIC), adjusted BIC (aBIC), and entropy. The lower the AIC, BIC, and aBIC are, the better the model fit [44]. Entropy ranges from 0 to 1; the closer the value is to 1, the more accurate the model classification is, and it is generally believed that values of 0.80 or greater indicate that the classification accuracy of the model reaches 90% [45]. The Lo-Mendell-Rubin likelihood ratio test (LMRT) and the bootstrapped likelihood ratio test (BLRT) were used to compare the fit of 2 adjacent models, where low and significant *P* values suggest a *k*-class model is superior to *k-1−*class model [46]. To visualize the latent profiles, the estimated class-specific means from the Mplus LPA model output were exported and plotted using line charts in Microsoft Excel version 16.1.

Second, latent profiles were named and assigned values to classify groups. Descriptive statistics were analyzed using SPSS (version 26.0, IBM Corp) to understand the data distribution characteristics of the profiles. Variables that conformed to a normal distribution were described as mean (SD), and comparisons between groups were made using 1-way ANOVA. Count data were described as frequency and percentage. Differences in sociodemographic and health-related factors between groups were assessed by chi-square tests. Finally, multivariate logistic regression analyses were performed with latent profiles of IC as the dependent variable and variables that were statistically significant in the univariate analyses as the independent variables. To avoid modeling errors, dummy variables were used for categorical data. The impact of the predictor variables and their statistical significance were assessed through regression coefficients (*β*), *P* values, odds ratios (ORs), and 95% CIs. A *P* value <.05 indicated a statistically significant difference.

### Qualitative Phase

#### Setting and Participants

A naturalistic, descriptive qualitative design is well suited to mixed methods research [47], enabling older adults to provide a rich and direct description of the goals, needs, and concerns of digital monitoring regarding their IC level. Stratified purposive sampling based on the categorical results of the LPA ensured the representation of participants originating from different IC profiles. Given the sequential continuity of the mixed methods study, the inclusion and exclusion criteria established for the quantitative phase were likewise applied to this phase. The first author contacted the older adults who had accepted the invitation to be interviewed during the quantitative phase, while explaining the purpose, content, and methodology of the further study and setting the time and place of the interviews.

#### Data Collection

Semistructured interviews were conducted between October and December 2024 to collect data. In accordance with the objectives of the study and the literature review [48], the research team developed the following interview outline: (1) “What is your idea of IC?” (2) “How would you evaluate your IC? Can you share some stories from your daily life where you have felt IC come into play or decline?” (3) “What do you think about using digital health tools for IC monitoring?” (4) “What do you hope the use of digital health tools for IC monitoring will do for you?” (5) “Do you have any concerns and doubts about using digital health tools for IC monitoring?” and (6) “Do you have any suggestions or expectations regarding the digital monitoring of IC?”

The first author reviewed the results of the quantitative phase of the IC assessment with the participants before the commencement of the interviews. Participants were interviewed in relaxed and comfortable parks, cafes, and clinic rooms. A total of 2 additional individual interviews were conducted via voice calls on the Tencent Conference platform. Each interview, led by the first author and assisted by another author for observation and field notes, lasted 20 to 68 minutes. Audio interviews were transcribed into text within 24 hours using Lark intelligent transcription. The first 2 authors (ZX and YN) and 2 qualitative experts (QX and YW) proofread the transcript and verified its contextual integrity. Data collection and analysis were synchronized in this study. Recruitment of participants was stopped when no further significant new information emerged, implying data saturation [49].

#### Data Analysis

The qualitative phase of data analysis was conducted with the main aim of obtaining the needs, intentions, and challenges of older adults regarding digital monitoring originating from the different IC profiles. Inductive combined with deductive content analysis was used to hand code the data to compare and integrate the similarities and differences between the different profiles horizontally [50]. Initially, the research team repeatedly reviewed interview transcripts to gain a holistic understanding of the data. Second, after interviewing 2 participants from each latent profile, the authors conducted open coding, assigning descriptive codes to meaningful text segments to develop a preliminary coding framework for guiding the remaining transcripts. Next, data from the same latent profiles were clustered and extracted contemporaneously by the first 2 authors (ZX and YN) following the attributes in the coding framework, which enabled the creation of customized personas. The framework was continuously refined and clarified throughout this process as new insights emerged or existing categories required redefinition. The final version of the framework constituted the thematic results. To capture the group-level commonalities within each persona, the primary author noted interview logs after each interview. These logs documented the author’s immediate impressions and holistic understanding of the participants, which later played a pivotal role in identifying the shared attributes that define each persona. Finally, the research team collaborated to define and describe the customized personas, outlining the participants’ differing attitudes, goals, needs, and available support that ultimately formed the subthemes for the 3 customized personas. Discrepancies between the first 2 authors (ZX and YN) during data analysis were addressed by an expert in qualitative nursing research to achieve harmonization of the results.

### Data Integration Procedure

As mentioned above, this study achieved methodological integration in that a quantitative LPA identified subgroups of IC that guided the qualitative phase of cluster sampling. Furthermore, the joint statistical and thematic presentation was used as a visualization tool to integrate and cross corroborate the main quantitative and qualitative findings at the level of results interpretation [51], providing a reference for future digital personalized interventions. The integrated customized personas were described jointly by the first 2 authors and iteratively discussed and revised in regular meetings of the research team, and finally confirmed by all authors.

### Ethical Considerations

This study was undertaken in accordance with the Declaration of Helsinki and approved by the Ethics Committee of Capital Medical University (approval number Z2023SY138). All participants enrolled in this study voluntarily and signed informed consent before the formal start of the assessment after being informed that they could withdraw at any time. Each older adult who participated in the quantitative survey received a gift worth approximately RMB 20 (US $2.81; a currency exchange rate of RMB 1≈US $0.1403 is applicable), while those who participated in the interviews received an additional gift worth RMB 40 (US $5.58). All data, audio recordings, and written records are stored on a password-protected laptop and backed up in the cloud; only the research team has access to them. In addition, all data were used for research purposes only and could not be disclosed without the participants’ consent. To ensure privacy protection, the participants’ personal information was anonymized and labeled “P1-P25.”

## Results

### Quantitative Phase

#### Participant Characteristics

A total of 481 older adults, aged 60-97 years, with a mean age of 70.7 (SD 6.8) years, participated in the quantitative phase. The results in Table 1 show that most participants were female (320/481, 66.5%), aged 60-69 years (234/481, 48.6%), currently married (356/481, 74%), and living in a married couple’s household (224/481, 46.6%). Most participants had a secondary education (318/481, 66.1%), a per capita monthly household income of RMB 3000-RMB 5999 (260/481, 54.1%), and engaged in light labor (241/481, 50.1%) before retirement. In addition, the majority of older adults rated their health as “very good or good” (258/481, 53.6%) and had 1 to 3 chronic diseases (323/481, 67.2%).

**Table 1:** Sociodemographic characteristics and univariate analysis of latent profiles (N=481).

Sociodemographic characteristics			Total, n (%)		IC^a^ impairment patterns, n (%)								Chi-square (*df*)			*P* value
					Profile 1 (n=92)^b^		Profile 2 (n=140)^c^		Profile 3 (n=249)^d^							
**Demographic variables**																
	**Sex**												3.0 (2)			.22
		Male		161 (33.5)		29 (31.5)		55 (39.3)		77 (30.9)						
		Female		320 (66.5)		63 (64.5)		85 (60.7)		172 (69.1)						
	**Age (years)**												47.2 (4)			<.001
		60-69		234 (48.6)		35 (38)		53 (37.9)		146 (58.6)						
		70-79		190 (39.5)		35 (38)		59 (42.1)		96 (38.6)						
		≥80		57 (11.9)		22 (23.9)		28 (20)		7 (2.8)						
	**Marital status**												8.2 (2)			.02
		Currently married		356 (74)		62 (67.4)		96 (68.6)		198 (79.5)						
		Divorced, widowed, or unmarried		125 (26)		30 (32.6)		44 (31.4)		51 (20.5)						
	**Family structures**												6.2 (4)			.19
		Lives alone		79 (16.4)		20 (21.7)		27 (19.3)		32 (12.9)						
		A married couple’s household		224 (46.6)		44 (47.8)		60 (42.9)		120 (48.2)						
		Multigeneration household		178 (37)		28 (30.4)		53 (37.8)		97(39)						
**Socioeconomic variables**																
	**Education level**												65.4 (6)			<.001
		Primary and below		85 (17.7)		33 (35.9)		38 (27.1)		14 (5.6)						
		Junior high school		163 (33.9)		30 (32.6)		49 (35)		84 (33.7)						
		Senior high school		155 (32.2)		23 (25)		29 (20.7)		103 (41.4)						
		College and above		78 (16.2)		6 (6.5)		24 (17.1)		48 (19.3)						
	**Type of preretirement labor force**												20.6 (4)			<.001
		Mental labor		149 (31)		22 (23.9)		45 (32.1)		82 (32.9)						
		Light labor		241 (50.1)		39 (42.4)		66 (47.1)		136 (54.6)						
		Heavy labor		91 (18.9)		31 (33.7)		29 (20.7)		31 (12.4)						
	**Monthly per capita household income (RMB)^e^**												52.6 (6)			<.001
		<1000		56 (11.6)		24 (26.1)		22 (15.7)		10 (4)						
		1000-2999		85 (17.7)		21 (22.8)		29 (20.7)		35 (14.1)						
		3000-5999		260 (54.1)		41 (44.6)		59 (42.1)		160 (64.3)						
		≥6000		80 (16.6)		6 (6.5)		30 (21.4)		44 (17.7)						
**Health-related variables**																
	**Self-rated health**												75.2 (8)			<.001
		Very good		61 (12.7)		4 (4.3)		11 (7.9)		46 (18.5)						
		Good		197 (41)		18 (19.6)		53 (37.9)		126 (50.6)						
		General		169 (35.1)		45 (48.9)		58 (41.4)		66 (26.5)						
		Poor		40 (8.3)		17 (18.5)		14 (10)		9 (3.6)						
		Very poor		14 (2.9)		8 (8.7)		4 (2.9)		2 (0.8)						
	**Number of chronic diseases**												25.8 (4)			<.001
		0		99 (20.6)		10 (10.9)		22 (15.7)		67 (26.9)						
		1-3		323 (67.2)		60 (65.2)		100 (71.4)		163 (65.5)						
		≥4		59 (12.3)		22 (23.9)		18 (12.9)		19 (7.6)						

^a^IC: intrinsic capacity.

^b^Percentage of the total=19.1 (92/481).

^c^Percentage of the total=29.1 (140/481).

^d^Percentage of the total=51.8 (249/481).

^e^A currency exchange rate of RMB 1≈US $0.1403 is applicable.

#### LPA Results

The scores in the 5 domains of IC were used as exogenous variables to model the latent profiles. The *z* score was used to eliminate the effects of different magnitudes on data analysis during data preprocessing. The fitting statistics for modeling 1-5 latent profiles sequentially are shown in Table 2. After comprehensive consideration of the latent profile metrics and clinical significance, we identified the 3-profile model as the best option for the following reasons: (1) the values of AIC, BIC, and aBIC decreased gradually as the number of profile subgroups increased, but beginning with the 4-profile model, the LMRT was greater than .05 and the minimum classification probability was less than 10%, signifying that this category may be spurious; (2) the LMRT and BLRT results were statistically significant (*P*<.05) for the 3-profile model, indicating that it was better than the 2-profile solution; and (3) the entropy value of the 3-profile model exceeded 0.8, indicating good model accuracy. Therefore, the 3-profile model was selected as the most appropriate model. To verify the reliability of the LPA, we calculated the average posterior probabilities for the 3 profiles. The results are shown in Multimedia Appendix 3, where the posterior probabilities were greater than 0.8, indicating that the model results for the 3 profiles were well separated [52].

**Table 2:** Latent profile analysis model fit of intrinsic capacity impairment patterns.

Model	Log(L)^a^	AIC^b^	BIC^c^	aBIC^d^	Entropy	LMRT^e^	BLRT^f^	Classification probability
1	–3409.625	6839.251	6881.010	6849.271	—^g^	—	—	1.00
2	–3085.278	6202.556	6269.370	6218.588	1.000	.2054	<.001	0.291/0.709
3	–2988.495	6020.991	6112.860	6043.034	0.906	.0174	<.001	0.191/0.291/0.518
4	–2946.724	5949.448	6066.372	5977.503	0.918	.1141	<.001	0.089/0.102/0.291/0.518
5	–2928.674	5925.348	6067.327	5959.414	0.920	.1226	<.001	0.046/0.080/0.080/0.291/0.503

^a^Log(L): Log-likelihood.

^b^AIC: Akaike information criterion.

^c^BIC: Bayesian information criterion.

^d^aBIC: adjusted BIC.

^e^LMRT: Lo-Mendell-Rubin likelihood ratio test.

^f^BLRT: bootstrapped likelihood ratio test.

^g^Not applicable.

#### Characteristics and Naming of Latent Profiles

**Figure 2:**
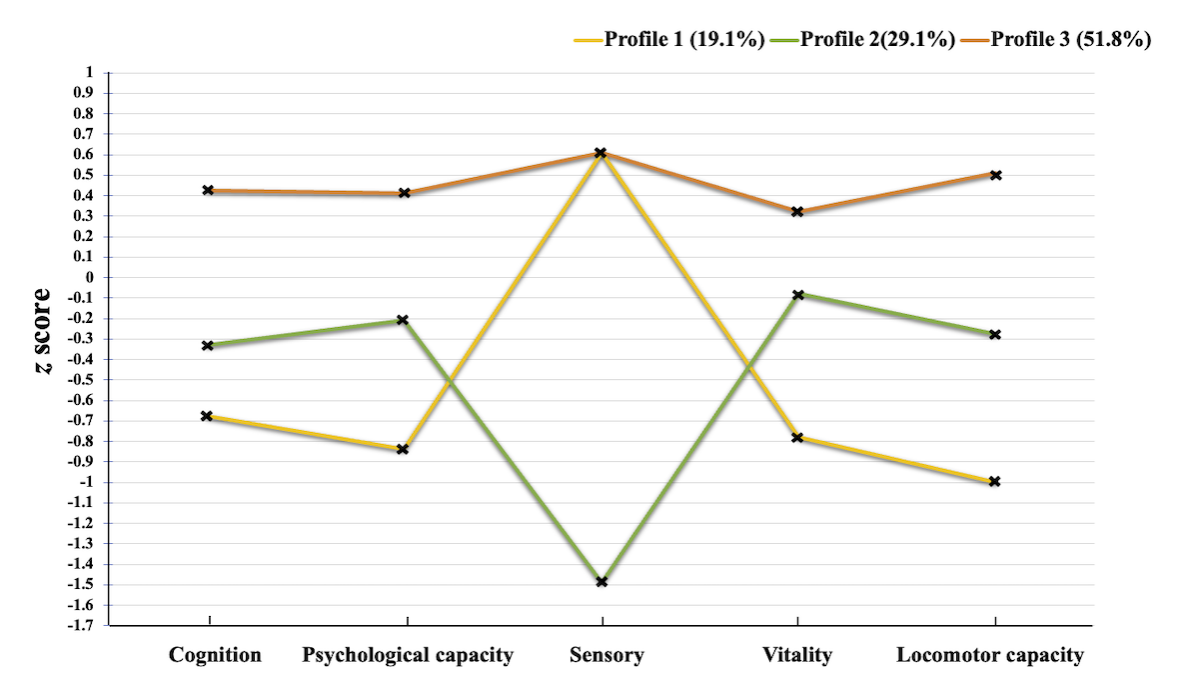
Characteristic distribution of 3 latent profiles. Profile 1: multisubdomain decline–IC imbalance group; profile 2: multisubdomain moderate–sensory deficit group; and profile 3: multisubdomain robust–whole balance group.

Figure 2 illustrates a graphical presentation of the *z* score for the 5 domains of IC in the 3 impairment profiles. Each profile was named according to its characteristics. Profile 1, constituting 19.1% (92/481) of the sample, was designated the “multisubdomain decline–IC imbalance group.” The most obvious characteristic of this group was the overall decline in IC, while only the sensory domain remained at a high level, resulting in an imbalance in IC. It should be noted that higher scores on the psychological domain indicated reduced psychological capacity. Profile 2, the “multisubdomain moderate–sensory deficit group,” included 29.1% (140/481) of the participants. This group showed a moderate IC level that was higher than those in profile 1 and was characterized by significant sensory deficits, reflecting a pattern of decline with single deficits. Profile 3 was the most prevalent in the sample, accounting for 51.8% (249/481) of cases. It was designated the “multisubdomain robust–whole balance group” owing to its exceptional performance in IC, with no significant loss of ability.

#### Comparison of Predictive Variables in Each Latent Profile

As shown in Table 1, the findings derived from the univariate analyses provide insights into the differences among the 3 latent profiles by using sociodemographic and health-related factors as predictor variables. Among these variables, age, marital status, education level, type of labor force prior to retirement, per capita monthly household income, self-rated health, and number of chronic diseases differed significantly (*P*<.05) among the 3 IC impairment patterns. No statistically significant differences were observed in sex and family structure.

Only variables that were statistically different between the IC subgroups in the univariate analyses were included in the multivariate logistic regression analyses (Table 3). Using profile 3, the “multisubdomain robust–whole balanced group,” as the reference group, older adults aged 80 years and older are more frequently found in either profile 1 (OR 9.190, 95% CI 2.986-28.284; *P*<.001) or profile 2 (OR 7.018, 95% CI 2.559-19.247, *P*<.001). As detailed in Table 3, as education levels rose, older adults were less likely to be categorized in profile 1 or profile 2. Those with a per capita monthly household income of ≥RMB 6000, indicating a higher quality of life and social status, were less likely to be attributed to profile 1 (OR 0.163, 95% CI 0.040-0.670; *P*<.05). Those with better self-rated health were more likely to belong to profile 3. For example, the probability of older adults rating their self-health as “very good” joining profile 1 (OR 0.037, 95% CI 0.005-0.265; *P*<.001) and profile 2 (OR 0.115, 95% CI 0.017-0.773; *P*<.05) was reduced. Older adults with no chronic diseases were less likely to be enrolled in profile 1 (OR 0.315, 95% CI 0.105-0.943; *P*<.05). The results, when viewed through profile 2 as the reference group, indicated a lower possibility of older adults with a per capita monthly household income ≥RMB 6000 (OR 0.219, 95% CI 0.061-0.782; *P*<.05) and self-rated health of “good” (OR 0.236, 95% CI 0.060-0.935; *P*<.05) belonging to profile 1.

**Table 3:** Multivariate logistic regression analysis of predictor variables of 3 latent profiles (N=481).

Predictor variables				Profile 1^a^ vs profile 3^b^				Profile 2^c^ vs profile 3				Profile 1 vs profile 2		
				*β* (SE)		OR^d^ (95% CI)		*β* (SE)		OR (95% CI)		*β* (SE)		OR (95% CI)
**Demographic variables**														
	**Age (years)**													
		60-69 (ref^e^)	N/A^f^		N/A		N/A		N/A		N/A		N/A	
		70-79	–0.079 (0.350)		0.924 (0.465-1.833)		0.119 (0.272)		1.126 (0.661-1.919)		–1.198 (0.353)		0.820 (0.410-1.639)	
		≥80	2.218 (0.574)^g^		9.190 (2.986-28.284)		1.949 (0.515)^g^		7.018 (2.559-19.247)		0.270 (0.445)		1.309 (0.548-3.130)	
	**Marital status**													
		Currently married (ref)	N/A		N/A		N/A		N/A		N/A		N/A	
		Divorced, widowed, or unmarried	–0.076 (0.352)		0.927 (0.465-1.847)		–0.162 (0.289)		0.850 (0.482-1.498)		0.086 (0.341)		1.090 (0.558-2.128)	
**Socioeconomic variables**														
	**Education level**													
		Primary and below (ref)	N/A		N/A		N/A		N/A		N/A		N/A	
		Junior high school	–1.004 (0.492)^h^		0.366 (0.140-0.961)		–1.041 (0.429)^h^		0.353 (0.152-0.819)		0.036 (0.422)		1.037 (0.454-2.371)	
		Senior high school	–1.484 (0.549)^i^		0.227 (0.077-0.665)		–1.899 (0.481)^g^		0.150 (0.058-0.384)		0.415 (0.510)		1.514 (0.557-4.117)	
		College and above	–1.755 (0.713)^h^		0.173 (0.043-0.699)		–1.241 (0.536)^h^		0.289 (0.101-0.827)		–0.514 (0.679)		0.598 (0.158-2.263)	
	**Type of preretirement labor force**													
		Mental labor (ref)	N/A		N/A		N/A		N/A		N/A		N/A	
		Light labor	–0.441 (0.392)		0.644 (0.299-1.387)		–0.295 (0.305)		0.744 (0.409-1.353)		–0.145 (0.406)		0.865 (0.391-1.915)	
		Heavy labor	0.218 (0.491)		1.244 (0.475-3.260)		–0.238 (0.420)		0.788 (0.346-1.797)		0.465 (0.483)		1.578 (0.612-4.071)	
	**Monthly per capita household income (RMB)** ^j^													
		<1000 (ref)	N/A		N/A		N/A		N/A		N/A		N/A	
		1000-2999	–0.558 (0.567)		0.572 (0.189-1.737)		–0.151 (0.529)		0.860 (0.305-2.424)		–0.407 (0.463)		0.665 (0.268-1.650)	
		3000-5999	–1.056 (0.538)		0.348 (0.121-0.999)		–0.780 (0.503)		0.458 (0.171-1.230)		–0.276 (0.448)		0.759 (0.315-1.825)	
		≥6000	–1.813 (0.721)^h^		0.163 (0.040-0.670)		–0.295 (0.570)		0.744 (0.243-2.275)		–1.518 (0.649)^h^		0.219 (0.061-0.782)	
**Health-related variables**														
		**Self-rated health**												
		Very good	–3.290 (1.000)^i^		0.037 (0.005-0.265)		–2.166 (0.974)^h^		0.115 (0.017-0.773)		–1.124 (0.882)		0.325 (0.058-1.833)	
		Good	–3.408 (0.886)^i^		0.033 (0.006-0.188)		–1.964 (0.923)^h^		0.140 (0.023-0.858)		–1.444 (0.703)^h^		0.236 (0.060-0.935)	
		General	–2.065 (0.871)^h^		0.127 (0.023-0.699)		–1.304 (0.924)		0.271 (0.044-1.661)		–0.761 (0.682)		0.467 (0.123-1.778)	
		Poor	–0.665 (0.952)		0.514 (0.080-3.323)		–.0.514 (1.004)		0.598 (0.084-4.278)		–0.151 (0.751)		0.860 (0.197-3.748)	
		Very poor (ref)	N/A		N/A		N/A		N/A		N/A		N/A	
	**Number of chronic diseases**													
		0	–1.155 (0.559)^h^		0.315 (0.105-0.943)		–0.413 (0.477)		0.661 (0.260-1.684)		–0.741 (0.539)		0.477 (0.166-1.370)	
		1-3	–0.357 (0.430)		0.700 (0.301-1.626)		0.195 (0.411)		1.215 (0.543-2.718)		–0.552 (0.391)		0.576 (0.268-1.239)	
		≥4 (ref)	N/A		N/A		N/A		N/A		N/A		N/A	

^a^Profile 1: multisubdomain decline–IC imbalance group.

^b^Profile 3: multisubdomain robust–whole balance group.

^c^Profile 2: multisubdomain moderate–sensory deficit group.

^d^OR: odds ratio.

^e^ref: reference category

^f^N/A: not applicable.

^g^*P*<.001.

^h^*P*<.05.

^i^*P*<.01.

^j^A currency exchange rate of RMB 1≈US $0.1403 is applicable.

#### Outcome Variables of Latent Profiles

As demonstrated in Table 4, there are significant differences in activities of daily living, frailty, and social frailty among the identified latent profiles of IC impairment. Subsequent post hoc comparisons revealed a gradual decline in functioning for latent profiles 3, 2, and 1, using the 3 functional abilities as the outcome variables.

**Table 4:** Effect of the 3 latent profiles on outcome variables (N=481).

Outcome variables (score range)	IC^a^ impairment patterns, mean (SD)				Post hoc comparison		*F* test (*df*)		*P* value
	Profile 1^b^ (n=92)	Profile 2^c^ (n=140)	Profile 3^d^ (n=249)						
BI^e^ (0-100)	88.70 (1.801)	93.32 (1.239)	99.20 (0.168)	3>2>1		33.866 (2, 478)		<.001	
FFP^f^ (0-5)	1.82 (0.105)	1.35 (0.094)	0.49 (0.043)	3>2>1		90.299 (2, 478)		<.001	
HALFT scale (0-5)	1.71 (0.126)	1.19 (0.103)	0.51 (0.049)	3>2>1		52.867 (2, 478)		<.001	

^a^IC: intrinsic capacity.

^b^Profile 1: multisubdomain decline–IC imbalance group.

^c^Profile 2: multisubdomain moderate–sensory deficit group.

^d^Profile 3: multisubdomain robust–whole balance group.

^e^BI: Barthel index.

^f^FFP: Fried frailty phenotype.

### Qualitative Phase

#### Interview Sample Characteristics

A total of 25 older adults volunteered to participate in the interviews, including 5 in profile 1, 10 in profile 2, and 10 in profile 3. Most participants were female (17/25, 68%). All participants had a mean age of 70.1 (SD 7.03, range 60-85) years, and had experienced between 0 and 4 IC domain declines. Detailed information is provided in Multimedia Appendix 4.

#### Developing Customized Personas

**Figure 3:**
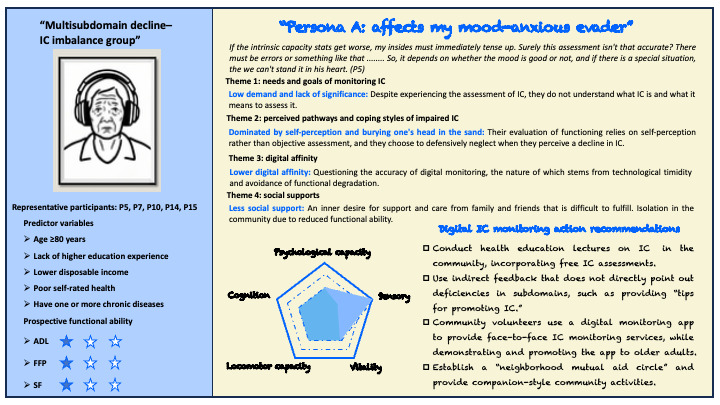
Persona A: affects my mood—anxious evader.

**Figure 4:**
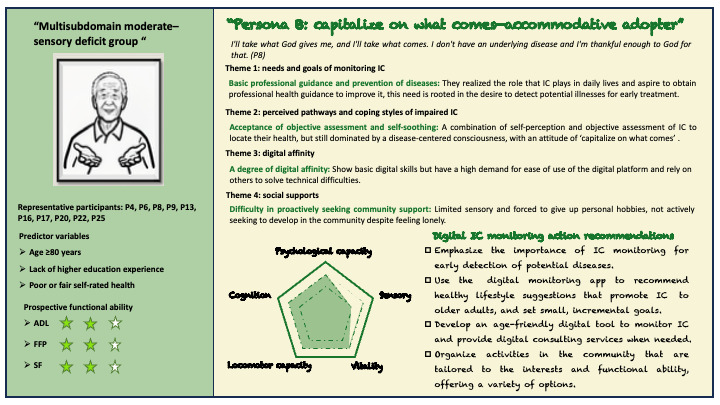
Persona B: capitalize on what comes—accommodative adopter.

**Figure 5:**
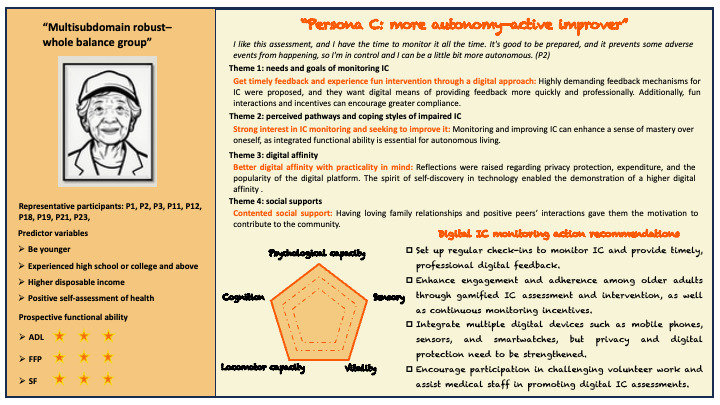
Persona C: more autonomy—active improver.

A total of 4 themes emerged in this study. In the hierarchical data analysis, 3 customized persons corresponding to different IC profiles were depicted, and subthemes under the themes that matched the persons were illustrated in Figures 3-5. Representative quotations were translated into English by the authors and confirmed by back-translation. In this section, the findings are reported as an integration of customized personas as follows.

#### Persona A: Affects My Mood—Anxious Evader

Narrative persona: affects my mood—anxious evader are female and belong to profile 1. They are older than those in other profiles, have lower education and disposable income, rate their health negatively, and are more likely to be chronically ill. Insights from the interviews suggest that they are psychologically fragile and stay away from anything that may bring about emotional stress. Moreover, the lack of social support for persona A, as evidenced by inadequate family care, a desire for companionship, and a neglected position in social groups, may further diminish their physical and psychological capacity in the face of potential stress. In addition, they were skeptical about using digital technology to assess IC and reluctant to ask for help based on past experiences. As a result, persona A showed less interest in digital monitoring of IC, which may have been a psychological coping mechanism that prompted the adoption of an avoidance strategy.

It certainly can't improve, this time this way, next time do better than this time, I don't think it can be done, people can only get older and worse.

P15

I know all about it, the results of this assessment are pretty much the same as how I feel about myself, the assessment is not very useful, and I'm doing enough to build muscle at home.

P10

I can type, but it might be difficult for other older adults. If I ask my child to help, she always tells me to take a break and not mess around. So, um..., but I don't want people to think I'm stupid if you ask for help, I'd be embarrassed and stressed.

P14

I used to be the lead dancer of the dance team, I thought I could still teach people how to dance when I got better, but I didn't realize that now I'm the most insignificant person in the dance team, and I can't dance .......I wish someone would tell me how to face society.

P7

#### Persona B: Capitalize on What Comes—Accommodative Adopter

Narrative persona: older adults in persona B, derived from profile 2, have a slight decline in IC, characterized by an impairment in sensory function. Compared to persona C, older adults in persona B were older, less educated, and more likely to rate their health as neutral or slightly negative. Participants expressed a desire to receive assistance from IC in the identification of potential illnesses and the acquisition of professional guidance, which was a passive strategy for treating illnesses. The acceptable attitude they displayed was derived from the Chinese proverb “capitalize on what comes,” which implied that since illness is unavoidable, it should be accepted calmly. Seemingly, this was not consistent with the Healthy Aging Framework, which posited the maintenance of IC to promote functional ability and quality of life. Moreover, they were willing to take advantage of digital health technology to assess IC but were marginally deficient in digital literacy, resulting in greater demands for functionality and ease of use. Regarding social support, they reported feeling cared for by family and friends. However, the activities and hobbies that they were compelled to abandon due to declining functionality also engendered helplessness and isolation, echoing the findings that they may be in the prefrailty and presocial frailty stages.

I'd like to know, what I should eat, and what I should watch out for to make me cognitively aware if I've lost my ability in a certain area.

P8

The disease is inevitable, no one will not be sick, old age is sicker, this is also in the natural, normal. Of course, when things go wrong, maybe it's just a minor problem, who doesn't do that in life? I can still sing, my head is not confused, and I don't have any trouble recognizing people, so I'm very satisfied with myself.

P6

In any case, it should be simplified. Technology is something that makes things simpler, it is 1 + 1 = 2, but why can't some older adults accept it? That's why I think it should be simplified; too many functions don't match the energy of the older adults.

P13

I have to give up a lot of activities and interests because of declining physical abilities.……The last six months I've been feeling really lonely and unenergetic. When I get up early, I go out and sit for two hours, and then the afternoon is particularly torturous.

P16

#### Persona C: More Autonomy—Active Improver

Narrative persona: participants in persona C are derived from profile 3 and represent older adults with high levels of IC. Compared to personas A and B, they are likely to be younger, more educated, have better disposable income, and a more positive assessment of their health. They showed a high level of interest in monitoring IC and were keen to maintain communication with their community health care team through age-friendly digital platforms for regular reminders, feedback on professional advice, and facilitation of functional improvement. Older adults in the technology-rich exploratory persona C also needed fun interventions and motivational approaches to help improve monitoring adherence, confidence, and sense of achievement. They suggested that free and community outreach is essential to increase access to IC monitoring. In addition, the interviews revealed that they were more likely to live in well-functioning family relationships and maintained good relationships with neighbors and peers through developing shared hobbies and were more autonomous and socially active.

After all, it needs to be assessed regularly, so could you design some mini-games? Design mini-games that exercise cognitive and finger dexterity and are similar to completing tasks. Older adults enjoy playing games just as much as children do.

P12

I think vitality, cognition, psychology and so on make up a state of equilibrium, and good IC is the most appropriate balance. For example, sometimes we may encounter some things, and our mental ability may be reduced, but we can regulate and restore it through some methods.

P23

Some of the digital stuff reflected ...... Actually, to the point where we use it quite simple. I have no difficulty at all, may have something to do with my previous work exposure to digital technology earlier.

P3

I often participate in social activities and volunteer, and I want to do what I can. I have been instructing residents on rubbish sorting and participating in community work, which I am willing to do as long as I have enough time.

P11

## Discussion

### Principal Findings

This study used an explanatory sequential mixed methods design to elucidate the data-driven link between heterogeneous IC profiles and psychological intentions toward digital IC monitoring in older adults. Specifically, an explanatory sequential mixed methods design was used, in which quantitative LPA provided the sampling framework and identified group-centered persona predictors. This informed subsequent qualitative interviews, which uncovered the underlying psychosocial narratives and specific digital monitoring intentions unique to each persona. “Persona A: affects my mood—anxious evader,” “persona B: capitalize on what comes—accommodative adopter,” and “persona C: more autonomy—active improver” represent differentiated IC characteristics among older adults and their digital monitoring intentions. These findings provide person-centered typology that can inform the development of tailored digital monitoring strategies within community-based integrated care for older adults.

While digital health tools offer efficient ways to monitor IC, it is vital that implementation strategies remain cautious and respect older adults’ willingness for digital engagement [29]. This caution is particularly pertinent to the “persona A: affects my mood—anxious evader” in this study, who was revealed to have the lowest level of IC and a negative willingness to engage in digital monitoring. They exhibited a higher prevalence of individuals aged 80 years or older, with low levels of education, low income, and poor self-rated health compared to the other 2 personas, echoing results reported in other studies showing a clear correlation between sociodemographic determinants and significant decline in IC [34,53]. A scoping review suggested that the 3 key factors contributing to digital exclusion among older adults are sociodemographic, physiological, and psychological factors, resulting in “unable” and “unwilling” attitudes [17], which further validate the trait of technological timidity in persona A.

The Health Belief Model can also provide an indirect explanation for this phenomenon, whereby older adults recognize that a decline in IC poses risks to their functional capacity and quality of life, yet the perceived benefits of digital IC monitoring are overshadowed by perceived barriers, including technological complexity, technophobia, and threats to self-esteem. As far as psychological states were concerned, the interview findings revealed that older adults within persona A exhibit a strong sense of self-protection, which is manifested in their avoidance of confronting their actual IC level. They expressed anxiety about the implication that an IC decline reflects age-related functional ability lapse, a condition that signifies aging. This phenomenon may be partially explained by Guo et al [54], who claimed that self-neglecting older adults are more exposed to delayed health actions and mental health damage and have a multisubdomain impairment of IC. Furthermore, social participation and family relationships are potential ameliorative factors in the decline of IC [53]. Persona A yearns for familial care and community engagement, though concerns about self-esteem and public image hinder proactive action, resulting in unmet social needs such as reminders and companionship [55].

Therefore, intervention strategies for Persona A should still be initiated with traditional IC monitoring to build trust via stable, low-pressure collaborative relationships. Community care providers should conduct comprehensive assessments of older adults’ daily living conditions, home environments, and social networks to design tailored supportive measures, which may involve gradual exposure to digital IC assessment tools and the provision of accessible social activities.

Persona B are characterized as “capitalize on what comes—accommodative adopter,” who have made peace with the decline in IC and demonstrate a more pragmatic, disease-centered demand for digital monitoring. They believed that the IC could provide early warning of hidden diseases and guide them to seek timely medical treatment by obtaining professional advice from the community health care team. ICOPE, focused on monitoring IC, is a key practice in the shift from disease-centered to function-centered approaches to healthy aging [56]. It is an innovative model that not only facilitates the transformation of community health care models but also urges older adults to overcome the cognitive limitations of a disease-focused mindset.

However, older adults in persona B are subconsciously constrained by self-agism, whereby they perceive the decline in IC as inevitable, which may reduce their perceived benefits of digital IC monitoring and lead to passive technological application [57]. A further characteristic is a notable sensory deficit that may result in diminished self-efficacy during digital interactions. As one grows older, the sensory domain is where the prevalence of impaired IC is most evident [21]. A previous study confirmed that augmented digital access could help address unmet medical needs and increase use of preventive care [58]. As participants desired, digital platforms integrated with wearable devices for real-time monitoring, capturing metrics such as step speed and gait, may mitigate interaction challenges arising from visual impairment. Besides, a flat structure and clear interface design of the digital platform can reduce technological dependence and enhance adherence to long-term IC monitoring.

Therefore, while monitoring IC through digital platforms, it is also essential to recommend and track lifestyle factors such as nutrition, physical activity, and social engagement for persona B. These recommendations should be tailored rather than generic health advice to alleviate their concerns about disease risks and functional decline. By linking behaviors that support IC to the motivation of disease detection, digital monitoring of IC becomes more proactive.

Persona C is an active collaborator in the community integrated care team’s use of digital IC monitoring; hence, it is designated as “more autonomy—active improver.” They were characterized by relatively younger age, higher educational attainment, disposable income, and more positive self-assessments of health, in line with previous studies that found that older adults with these characteristics had better IC and lower risk of decline [59,60]. Older adults in this persona were particularly proactive in mastering their IC to seek autonomy in later life. They believed that maintaining self-management abilities is essential for older adults’ health and independence, especially in the face of IC decline, and that it also moderates the relationship between quality of life and physical function [33]. Moreover, persona C showed higher digital literacy, as evidenced by their constructive comments on the needs for digital platforms, such as timely and professional feedback and interesting incentives and interventions to stimulate adherence and engagement in health management. As confirmed by prior practical experience studies, timely feedback mechanisms and support networks enhanced interactions between older adults and community integrated care teams, providing an efficient interface for subsequent steps in ICOPE [14,15]. Additionally, another characteristic of this persona was that they fully perceived and used social support and further contributed to society to the best of their ability. Similar findings indicated that when older adults had a greater sense of control, they were able to actively use perceived social support to achieve goals they deemed important [61]. On the other hand, these older adults unintentionally accessed more resources or strategies while participating in social activities, creating a positive feedback loop for the improvement of IC [62].

Consequently, digital monitoring of persona C’s IC should incorporate motivational elements to encourage long-term engagement, thereby reinforcing a sense of competence and autonomy. To sustain engagement, the monitoring platform could incorporate gamification features such as progress tracking, challenge missions, and reward mechanisms for achieving IC-related goals, which could directly enhance a sense of accomplishment and confidence in self-management, aligning with their proactive approach to healthy aging.

### Strengths and Limitations

The primary contribution of this study is the application of a data-driven persona framework to elucidate older adults’ psychological intentions regarding digital IC monitoring. These personas synthesize quantitative predictors of IC profiles with qualitative insights into attitudes, goals, ability reserves, and support resources. Methodologically, the initial LPA established a sampling and analytical framework ensuring that interviews focused on revealing rich contextual narratives behind each statistically defined profile. This approach advances the discourse from describing subgroup characteristics to explaining psychological intentions, constructing personas that are both statistically grounded and vividly descriptive. Additionally, customized personas will inform targeted recruitment strategies and the development of IC monitoring systems oriented toward real abilities and goals, ensuring monitoring pathways align with the readiness and needs of diverse groups.

However, this study has several limitations. First, the participants in this study were recruited from urban communities in Beijing, China, and were selected through convenience sampling. Therefore, the findings may not be generalizable to older adults in rural or remote settings, where factors such as health care accessibility, digital literacy, socioeconomic status, and community support structures may present distinct profiles that could influence both IC trajectories and engagement with digital monitoring. Second, sex differences in the value of health and social engagement resulted in a majority of participants being female, which has an unknown effect on depicting persona characteristics. Third, the interview sample based on the results of the quantitative phase has differences in the number of participants, which can be explained by the poorer health and lower interest of older adults in profile 1. The final limitation is that the 3 customized personas provide only a momentary sketch of a specific population. To keep the personas up to date, future studies will address this by collecting additional data through follow-up visits.

### Conclusion

This study integrates quantitative feature analysis with qualitative narrative exploration to investigate the heterogeneous IC characteristics of older adults and their psychological intentions toward digital IC monitoring. Through a comprehensive review, 3 distinct IC profiles were identified, and corresponding customized personas were developed. These personas not only reflect the multidimensional nature of IC but also capture underlying attitudes, personal goals, digital affinity, and social support. The findings provide practical, persona-based tools for community health care providers to tailor communication, support, and technology adoption strategies, enhancing the acceptance and effectiveness of digital IC monitoring among older adults.

## Appendix

Multimedia Appendix 1 Good Reporting of a Mixed Methods Study (GRAMMS) checklist.

Multimedia Appendix 2 Instruments used to assess intrinsic capacity and functional ability.

Multimedia Appendix 3 Mean attribution probabilities for each latent profile.

Multimedia Appendix 4 Sociodemographic characteristics of interview participants (n=25).

## Abbreviations

aBIC: adjusted BIC
AIC: Akaike information criterion
BIC: Bayesian information criterion
BLRT: bootstrapped likelihood ratio test
GRAMMS: Good Reporting of a Mixed Methods Study
HEALTHY: Comprehensive Evaluation of Intrinsic Capacity in China Study
IC: intrinsic capacity
ICOPE: Integrated Care for Older People
LMRT: Lo-Mendell-Rubin likelihood ratio test
LPA: latent profile analysis
OR: odds ratio
WHO: World Health Organization

## Data Availability

The datasets generated or analyzed during this study are not publicly available due privacy or ethical restrictions, but are available from the corresponding author on reasonable request.

## References

[1] (2024). Integrated Care for Older People (ICOPE): Guidance for Person-Centred Assessment and Pathways in Primary Care. 2nd Edition.

[2] Goh SHE, Zhang D, Tan KH, Koh SLS (2025). Nurses' perception of their role in leading nurse-led interventions in intrinsic capacity assessment to improve nursing care of older adults. J Adv Nurs.

[3] Ahmadi S, Afshar PF, Malakouti K, Azadbakht M (2025). The relationship between intrinsic capacity and functional ability in older adults. BMC Geriatr.

[4] Beard JR, Jotheeswaran AT, Cesari M, Araujo de Carvalho I (2019). The structure and predictive value of intrinsic capacity in a longitudinal study of ageing. BMJ Open.

[5] (2019). Integrated Care for Older People (ICOPE): Guidance for Person-Centred Assessment and Pathways in Primary Care.

[6] Liu M, Zhang M, Zhou J, Song N, Zhang L (2023). Research on the healthy life expectancy of older adult individuals in China based on intrinsic capacity health standards and social stratification analysis. Front Public Health.

[7] Cao X, Yi X, Chen H, Tian Y, Li S, Zhou J (2024). Prevalence of intrinsic capacity decline among community-dwelling older adults: a systematic review and meta-analysis. Aging Clin Exp Res.

[8] Ma L, Chhetri JK, Zhang Y, Liu P, Chen Y, Li Y (2020). Integrated care for older people screening tool for measuring intrinsic capacity: preliminary findings from ICOPE pilot in China. Front Med (Lausanne).

[9] Zhou Y, Wang G, Li J, Liu P, Pan Y, Li Y, Ma L (2024). Trajectory of intrinsic capacity among community-dwelling older adults in China: the China Health and Retirement Longitudinal Study. Arch Gerontol Geriatr.

[10] Sánchez-Sánchez JL, Lu W, Gallardo-Gómez D, Del Pozo Cruz B, de Souto Barreto P, Lucia A (2024). Association of intrinsic capacity with functional decline and mortality in older adults: a systematic review and meta-analysis of longitudinal studies. Lancet Healthy Longev.

[11] Hwang AC, Chen LY, Tseng SH, Huang CY, Yen KH, Chen LK (2024). Intrinsic capacity transitions predict overall and cause-specific mortality, incident disability, and healthcare utilization. J Nutr Health Aging.

[12] Ma L (2025). Physical activity, sedentary behaviour, and intrinsic capacity at older ages: get active!. Lancet Healthy Longev.

[13] Berbon C, Rolland Y, Takeda C, Lafont C, Tavassoli N, De Kerimel J (2025). WHO ICOPE programme adherence of 8672 older age people over 2-years of follow-up. J Adv Nurs.

[14] Berbon C, Takeda C, Balardy L, Lafont C, Tavassoli N, Carrie I (2025). Implementing the WHO ICOPE program in clinical practice: three years of lessons from monitoring 27,082 participants using the ICOPE monitor digital tool. J Gerontol A Biol Sci Med Sci.

[15] Tavassoli N, de Souto Barreto P, Berbon C, Mathieu C, de Kerimel J, Lafont C (2022). Implementation of the WHO integrated care for older people (ICOPE) programme in clinical practice: a prospective study. Lancet Healthy Longev.

[16] Stara V, Soraci L, Takano E, Kondo I, Möller J, Maranesi E (2023). Intrinsic capacity and active and healthy aging domains supported by personalized digital coaching: survey study among geriatricians in Europe and Japan on eHealth opportunities for older adults. J Med Internet Res.

[17] Ge H, Li J, Hu H, Feng T, Wu X (2025). Digital exclusion in older adults: a scoping review. Int J Nurs Stud.

[18] Özgül E, Döner NH, Çürük GN (2025). Investigating older adults' technology attitudes: psychometric evaluation and cross-cultural adaptation of the TechPH scale. Int J Med Inform.

[19] Li X, Zhang N, Yang J, Geng Z, Zhou J, Zhang J (2024). Weight management personas of breast cancer patients undergoing chemotherapy in China: a multi-method study. BMC Med Inform Decis Mak.

[20] LeRouge C, Ma J, Sneha S, Tolle K (2013). User profiles and personas in the design and development of consumer health technologies. Int J Med Inform.

[21] Yu J, Si H, Jin Y, Qiao X, Ji L, Bian Y (2022). Patterns of intrinsic capacity among community-dwelling older adults: identification by latent class analysis and association with one-year adverse outcomes. Geriatr Nurs.

[22] Ma L, Zheng E, Fang Y, Chen H, Cai S, Luo F (2024). Intrinsic capacity loss rates and protective factors among individuals aged 80 years and older in Chinese nursing homes: a latent class analysis. Geriatr Nurs.

[23] Groos SS, Linn AJ, Kuiper JI, van Schoor NM, van der Velde N, van Weert JC (2024). Combining user-centered design and behavioral theory to enhance health technologies: a personas-based approach for a primary-care based multifactorial falls risk assessment tool. Int J Med Inform.

[24] Holden RJ, Kulanthaivel A, Purkayastha S, Goggins KM, Kripalani S (2017). Know thy eHealth user: development of biopsychosocial personas from a study of older adults with heart failure. Int J Med Inform.

[25] Yang Z, Xu L, Gao Y, Zhang C, Wang A (2025). Tailored personas for self-management in home-based cardiac rehabilitation for patients with coronary heart disease: a qualitative study. Int J Nurs Stud.

[26] Jolliff A, Hill J, Zuraw M, Elliott C, Werner NE (2024). Representing the needs of rural caregivers of people living with Alzheimer’s disease and related dementias through user personas. Innov Aging.

[27] Ivankova NV, Creswell JW, Stick SL (2006). Using mixed-methods sequential explanatory design: from theory to practice. Field Methods.

[28] Robinson P (2007). Designing and conducting mixed methods research. Aust N Z J Public Health.

[29] Schäfer K, Rasche P, Bröhl C, Theis S, Barton L, Brandl C (2019). Survey-based personas for a target-group-specific consideration of elderly end users of information and communication systems in the German health-care sector. Int J Med Inform.

[30] O'Cathain A, Murphy E, Nicholl J (2008). The quality of mixed methods studies in health services research. J Health Serv Res Policy.

[31] Chow SC, Shao J, Wang H, Lokhnygina Y (2017). Sample Size Calculations in Clinical Research. 3rd Edition.

[32] Nylund-Gibson K, Garber AC, Carter DB, Chan M, Arch DAN, Simon O (2023). Ten frequently asked questions about latent transition analysis. Psychol Methods.

[33] Liu Q, Li X, Hu M, Zhao Y, Wu S, Feng H (2024). Factors influencing the self-management ability among older adults experiencing intrinsic capacity decline: a cross-sectional study. Front Aging Neurosci.

[34] Yu S, Wang J, Xia Y, Tang Q (2024). The status quo and influencing factors of intrinsic capacity among community-dwelling older adults from the perspective of ecological systems theory: a cross-sectional study. BMC Geriatr.

[35] Mahoney FI, Barthel DW (1965). Functional evaluation: The Barthel index. Md State Med J.

[36] Fried LP, Tangen CM, Walston J, Newman AB, Hirsch C, Gottdiener J (2001). Frailty in older adults: evidence for a phenotype. J Gerontol A Biol Sci Med Sci.

[37] Ma L, Sun F, Tang Z (2018). Social frailty is associated with physical functioning, cognition, and depression, and predicts mortality. J Nutr Health Aging.

[38] Folstein MF, Folstein SE, McHugh PR (1975). "Mini-mental state". A practical method for grading the cognitive state of patients for the clinician. J Psychiatr Res.

[39] Rubenstein LZ, Harker JO, Salvà A, Guigoz Y, Vellas B (2001). Screening for undernutrition in geriatric practice: developing the short-form mini-nutritional assessment (MNA-SF). J Gerontol A Biol Sci Med Sci.

[40] Guralnik JM, Simonsick EM, Ferrucci L, Glynn RJ, Berkman LF, Blazer DG (1994). A short physical performance battery assessing lower extremity function: association with self-reported disability and prediction of mortality and nursing home admission. J Gerontol.

[41] Yesavage JA, Sheikh JI (2008). Geriatric depression scale (GDS). Clin Gerontol.

[42] Huang ZT, Lai ET, Luo Y, Woo J (2024). Social determinants of intrinsic capacity: a systematic review of observational studies. Ageing Res Rev.

[43] Leung CLK, Li K, Wei VWI, Tang A, Wong SYS, Lee SS (2022). Profiling vaccine believers and skeptics in nurses: a latent profile analysis. Int J Nurs Stud.

[44] Tofighi D, Enders CK, Hancock GR, Samuelsen KM (2007). Identifying the correct number of classes in growth mixture models. Advances in Latent Variable Mixture Models.

[45] Tein J, Coxe S, Cham H (2013). Statistical power to detect the correct number of classes in latent profile analysis. Struct Equ Modeling.

[46] Nylund KL, Asparouhov T, Muthén BO (2007). Erratum: deciding on the number of classes in latent class analysis and growth mixture modeling: a Monte Carlo simulation study (Struct Equ Modeling. 2007;14(4):535-569). Struct Equ Modeling.

[47] Sandelowski M (2000). Whatever happened to qualitative description?. Res Nurs Health.

[48] Möller J, Stara V, Amabili G, Barbarossa F, Riccardi GR, Martella C (2024). Toward innovation in healthcare: an analysis of the digital behavior of older people in Europe and Japan for the introduction of a technological coaching system. Healthcare (Basel).

[49] Rahimi S, Khatooni M (2024). Saturation in qualitative research: an evolutionary concept analysis. Int J Nurs Stud Adv.

[50] Hsieh HF, Shannon SE (2005). Three approaches to qualitative content analysis. Qual Health Res.

[51] Guetterman TC, Fetters MD, Creswell JW (2015). Integrating quantitative and qualitative results in health science mixed methods research through joint displays. Ann Fam Med.

[52] Weller BE, Bowen NK, Faubert SJ (2020). Latent class analysis: a guide to best practice. J Black Psychol.

[53] Zhao Y, Chen Y, Xiao LD, Liu Q, Nan J, Li X (2024). Intrinsic capacity trajectories, predictors and associations with care dependence in community-dwelling older adults: a social determinant of health perspective. Geriatr Nurs.

[54] Guo W, Meng L, Han J, Yang B, Sun J, Guo Y (2024). Intrinsic capacity and its association with predictors among Chinese empty nest older adults in communities: a latent class analysis. BMC Geriatr.

[55] Ciuffreda I, Amabili G, Casaccia S, Benadduci M, Margaritini A, Maranesi E (2023). Design and development of a technological platform based on a sensorized social robot for supporting older adults and caregivers: GUARDIAN ecosystem. Int J of Soc Robotics.

[56] Hoogendijk EO, Dent E, Koivunen K (2023). Intrinsic capacity: an under-researched concept in geriatrics. Age Ageing.

[57] Ishikawa M (2023). Internalization of negative societal views on old age into self-perceptions of aging: exploring factors associated with self-directed ageism. Front Sociol.

[58] Zhang Z, Yang L, Cao H (2026). Effect of inter-generational living arrangement and digital exclusion on unmet healthcare needs among older adults: findings from two national cohort studies. J Clin Nurs.

[59] Beard JR, Si Y, Liu Z, Chenoweth L, Hanewald K (2022). Intrinsic capacity: validation of a new WHO concept for healthy aging in a longitudinal Chinese study. J Gerontol A Biol Sci Med Sci.

[60] Wei X, Chen Y, Qin J, Yang Y, Yang T, Yan F (2024). Factors associated with the intrinsic capacity in older adults: a scoping review. J Clin Nurs.

[61] Yu J, Jin Y, Si H, Bian Y, Liu Q, Qiao X (2023). How does social support interact with intrinsic capacity to affect the trajectory of functional ability among older adults? Findings of a population-based longitudinal study. Maturitas.

[62] Liu M, Chang Y, Zhao S, Guo W, Ji X, Liu Y (2024). The effect of the interaction between intrinsic capacity and social support on the trajectories of activities of daily living in older adults. Geriatr Nurs.

