# Supplementary material for: Developing Customized Personas to Capture Intrinsic Capacity Profiles and Digital Monitoring Intentions in Older Adults: Mixed Methods Study
Source: JMIR Aging. 2026 May 27;9:e82867. doi: 10.2196/82867 (PMC13254505; doi:10.2196/82867)
Supplement: Multimedia Appendix 1 [file aging_v9i1e82867_app1.docx]

**Multimedia Appendix 1:** **Good Reporting of a Mixed Methods Study (GRAMMS) checklist**

| Guideline | Page information |
| --- | --- |
| 1. Describe the justification for using a mixed methods approach to the research question | In the last two paragraphs of the background |
| 1. Describe the design in terms of the purpose, priority and sequence of methods | In the study procedures |
| 1. Describe each method in terms of sampling, data collection and analysis | Quantitative and qualitative phases in the methods |
| 1. Describe where integration has occurred, how it has occurred and who has participated in it | Data integration procedure in the methods |
| 1. Describe any limitation of one method associated with the present of the other method | In the strengthens and limitations |
| 1. Describe any insights gained from mixing or integrating methods | In the results and discussion |

Reference: O’Cathain, A., Murphy, E., Nicholl, J., 2008. The quality of mixed methods studies in health services research. J Health Serv Res Policy 13, 92–98.
