# Supplementary material for: Developing Customized Personas to Capture Intrinsic Capacity Profiles and Digital Monitoring Intentions in Older Adults: Mixed Methods Study
Source: JMIR Aging. 2026 May 27;9:e82867. doi: 10.2196/82867 (PMC13254505; doi:10.2196/82867)
Supplement: Multimedia Appendix 2 [file aging_v9i1e82867_app2.docx]

**Multimedia Appendix 2:** **Instruments used to assess intrinsic capacity and** **functional ability.**

| Variables | Instruments | Score range | Dimensions and scoring criteria |
| --- | --- | --- | --- |
| Intrinsic Capacity |  | | |
| Cognition | MMSE^a^ | 0-30 | - Orientation (10 points)  1. Time Orientation (5 points): Questions about the current year, season, month, date, and day of the week. 2. Place Orientation (5 points): Questions about the current country, state/province, city, hospital/floor, and room number.  - Registration (3 points)  1. Immediate recall of three unrelated words repeated by the examiner.  - Attention and Calculation (5 points)  1. Serial subtraction of 7s starting from 100 (1 point per correct step).  - Recall (3 points)  1. Delayed recall of the three words from the Registration section.  - Language and Visuospatial (9 points)  1. Naming (2 points): Identifying common objects. 2. Repetition (1 point): Repeating a phrase (e.g., "No ifs, ands, or buts"). 3. Three-Stage Command (3 points): Following a verbal/physical instruction. 4. Reading (1 point): Reading and obeying a written command. 5. Writing (1 point): Writing a complete sentence.   11. Visual Construction (1 point): Copying two intersecting pentagons.  **Scores from this examination vary by age and education, but overall lower scores indicate poorer cognitive function.** |
| Psychological capacity | GDS-15^b^ | 0-15 | The GDS-15 includes 15 questions covering a wide range of emotional, behavioural, and somatic symptoms, but these questions are integrated into a single total score without the separate dimensions of the original design.  **Higher scores indicate more severe depressive symptoms.** |
| Sensory |  | 0-2 | **Sensory scores are cumulated from vision and hearing scores.** |
| Vision | WHO vision screening chart | 0-1 | To conduct this assessment, a space with good lighting and big enough to ensure the correct testing distance (e.g. 3 m for distance vision, 40 cm for near vision) is required. If the person already wears spectacles, examine visual acuity while wearing them.   - **Test distance vision**  1. Test each eye separately (monocularly), starting with the right eye first, then the left eye. 2. Use an occlude (or a hand) to cover the eye not being tested. 3. Verify that the person is looking at the chart straight, without turning or twisting their head. 4. Ensure the chart is 3 m from the person’s eyes. 5. Start with the large E. If at least two of the large E are seen, continue the test with the small E. 6. Record the results for the right eye, and repeat the steps for the left eye. 7. A pass result of the distance vision test is when each eye sees at least three of the small E.  - **Test near vision**  1. Test both eyes together (binocularly). 2. Hold the near chart at 40 cm from the person’s eyes. 3. A pass result of the near vision test is when both eyes see at least three of the E.   **If both distance and near vision pass the test, the visual acuity score is 1. Failure to pass either item is scored as 0.** |
| Hearing | Whisper voice test | 0-1 | The whisper voice test conducted in a quiet room to minimize background noise. Standing behind the patient (to block visual cues) and masking the non-test ear, a combination of three words or numbers is whispered at arm's length (two feet) from the test ear. Older adults were considered to have passed the test if they could correctly repeat all three words or numbers. If the answer was incorrect or not answered at all, the test was repeated again using a different three words or numbers. Overall, the older person was considered to have passed the test if they correctly repeated at least three of the possible six letters or numbers. The other ear is then assessed in a similar manner.  **Passing the test in both ears counts as a hearing score of 1. Failure to pass the test in both ears or passing in only one ear is scored as a 0.** |
| Vitality | MNA-SF^c^ | 0-14 | - Food Intake Decline (0–2 points)   Evaluates reduced dietary intake over the past 3 months.  0 points: Severe decrease; 1 point: Moderate decrease; 2 points: No decrease.   - Weight Loss (0–3 points)   Assesses unintentional weight loss in the past 3 months.  0 points: >3 kg lost; 1 point: 1–3 kg lost; 2 points: Unknown weight loss; 3 points: No weight loss.   - Mobility (0–2 points)   Reflects physical activity level.  0 points: Bedbound or severely impaired; 1 point: Can move but does not go out; 2 points: Goes out regularly.   - Psychological Stress or Acute Disease (0–2 points)   Accounts for recent psychological distress or acute illness.  0 points: Yes (in the past 3 months); 2 points: No.   - Neuropsychological Problems (0–2 points)   Screens for dementia, depression, or severe cognitive impairment.  0 points: Severe impairment; 1 point: Mild/moderate impairment; 2 points: No impairment.   - Body Mass Index (BMI) or Calf Circumference (0–3 points)   BMI scoring (if measured): 0 points: <19 kg/m²; 1 point: 19–21 kg/m²; 2 points: 21–23 kg/m²; 3 points: ≥23 kg/m².  Calf circumference (if BMI unavailable): 0 points: <31 cm; 3 points: ≥31 cm.  **Higher scores indicate better vitality.** |
| Locomotor capacity | SPPB^d^ | 0-12 | - Balance: Stand for 10 seconds with feet in each of the three positions. Use the sum of the three scores. If any not attempted, end balance tests. - Gait speed: time to walk 4 m   4 points: < 4.82 seconds; 3 points: 4.82–6.20 seconds; 2 points: 6.21–8.70 seconds; 1 points: > 8.70 seconds; Unable to complete 0 points   - Chair rise: time to rise from a chair five times   4 points: < 11.19 seconds 4 points; 3 points: 11.2–13.69 seconds; 2 points: 13.7–16.69 seconds; 1 point: 16.7–59.90 seconds; 0 points: > 60 seconds or unable to complete  **Higher scores indicate better locomotor capacity.** |
| Functional ability |  | | |
| Activities of daily living | Barthel Index | 0-100 | The Barthel Index assesses 10 key dimensions, with varying point allocations based on the level of independence.   - Feeding   10 points: independent (uses utensils without assistance); 5 points: needs help (e.g., cutting food); 0 points: fully dependent (e.g., tube-fed).   - Bathing   5 points: washes entire body independently (tub, shower, or bed bath); 0 points: requires assistance.   - Grooming   5 points: independent in washing face, brushing teeth, and combing hair; 0 points: needs assistance.   - Dressing   10 points: selects clothes and dresses/undresses independently (including shoes and fasteners); 5 points: needs partial help (e.g., buttons or zippers); 0 points: fully dependent.   - Bowel Control   10 points: full control (no accidents); 5 points: occasional accidents (≤1/week); 0 points: incontinent or requires manual evacuation.   - Bladder Control   10 points: full control or manages catheter independently; 5 points: occasional accidents (≤1/day); 0 points: incontinent or dependent on caregiver.   - Toilet Use   10 points: transfers to toilet, cleans self, and adjusts clothing independently; 5 points: needs assistance for transfers or cleaning; 0 points: fully dependent.   - Bed-to-Chair Transfer   15 points: independent (may use aids like handrails); 10 points: requires minor assistance (e.g., steadying); 5 points: needs major assistance (e.g., lifting by others); 0 points: unable to transfer.   - Mobility (Walking)   15 points: walks ≥50 meters independently (may use assistive devices); 10 points: needs supervision or minor assistance; 5 points: wheelchair-independent (self-propels); 0 points: immobile.   - Stair Climbing   10 points: climbs stairs independently (may use handrails or cane); 5 points: needs assistance or supervision; 0 points: unable to climb. |
| Frailty | Fried frailty phenotype | 0-5 | - Unintentional Weight Loss (1 point)   Criterion met: Self-reported unintentional weight loss of ≥5% of body weight in the past year.   - Weakness -Grip Strength (1 point)   Criterion met: Grip strength below sex- and BMI-adjusted thresholds (measured by dynamometer).   - Self-Reported Exhaustion (1 point)   Criterion met: Positive response to either of two statements: "I felt that everything I did was an effort", "I could not get going"   - Slow Walking Speed (1 point)   Criterion met: Time to walk 4 meters exceeds sex- and height-adjusted thresholds.   - Low Physical Activity (1 point)   Criterion met: Kilocalories expended per week fall below sex-specific thresholds.  **Higher scores indicate greater frailty.** |
| Social frailty | HALFT scale | 0-5 | The HALFT contains six questions on providing help to others, social activities, recreational activities, loneliness, daily expenses, and inner expression, but is not dimensioned. There are different scores based on frequency and degree.  **Higher scores indicate greater social frailty.** |

^a^MMSE: Mini-Mental State Examination.

^b^GDS-15: Geriatric Depression Scale-15.

^c^MHA-SF: Mini Nutritional Assessment Short-Form.

^d^SPPB: Short Physical Performance Battery test.
