# Supplementary material for: Developing Customized Personas to Capture Intrinsic Capacity Profiles and Digital Monitoring Intentions in Older Adults: Mixed Methods Study
Source: JMIR Aging. 2026 May 27;9:e82867. doi: 10.2196/82867 (PMC13254505; doi:10.2196/82867)
Supplement: Multimedia Appendix 3 [file aging_v9i1e82867_app3.docx]

**Multimedia Appendix 3:** **Mean attribution probabilities for each latent profile.**

| Latent profile | Profile 1^a^ | Profile 2^b^ | Profile 3^c^ |
| --- | --- | --- | --- |
| Profile 1^a^ | 0.882 | <0.001 | 0.118 |
| Profile 2^b^ | <0.001 | 1.000 | <0.001 |
| Profile 3^c^ | <0.001 | 0.037 | 0.963 |

^a^Profile 1: multi-subdomain recession-intrinsic capacity imbalance group.

^b^Profile 2: multi-subdomain moderate-sensory deficit group.

^c^Profile 3: multi-subdomain robust-whole balance group.
