# Supplementary material for: Developing Customized Personas to Capture Intrinsic Capacity Profiles and Digital Monitoring Intentions in Older Adults: Mixed Methods Study
Source: JMIR Aging. 2026 May 27;9:e82867. doi: 10.2196/82867 (PMC13254505; doi:10.2196/82867)
Supplement: Multimedia Appendix 4 [file aging_v9i1e82867_app4.docx]

**Multimedia Appendix 4:** **Sociodemographic characteristics of interviews’ participants (n=25).**

| **Characteristic** | **Frequency (n)** | **Percentage (%)** |
| --- | --- | --- |
| **Age (year)** |  |  |
| 60~70 | 15 | 60.00 |
| 71~80 | 9 | 36.00 |
| ＞80 | 1 | 4.00 |
| **Gender** |  |  |
| Male | 8 | 32.00 |
| Female | 17 | 68.00 |
| **Ethnicity** |  |  |
| Han | 23 | 92.00 |
| National minority | 2 | 8.00 |
| **Educational level** |  |  |
| Primary and below | 1 | 4.00 |
| Junior high school | 6 | 24.00 |
| Senior high school | 7 | 28.00 |
| College and above | 11 | 44.00 |
| **Marital status** |  |  |
| Currently married | 19 | 76.00 |
| Widowed/Unmarried | 6 | 24.00 |
| **Type of pre-retirement labor force** |  |  |
| Mental labor | 13 | 52.00 |
| Light labor | 9 | 36.00 |
| Heavy labor | 3 | 12.00 |
| **Monthly per capita household income (yuan^a^)** |  |  |
| 1000-2999 | 12 | 48.00 |
| 3000-5999 | 9 | 36.00 |
| ≥6000 | 4 | 16.00 |
| **IC^b^ impairment patterns** |  |  |
| Profile 1^c^ | 5 | 20.00 |
| Profile 2^d^ | 10 | 40.00 |
| Profile 3^e^ | 10 | 40.00 |
| **Self-rated health** |  |  |
| Very good | 5 | 20.00 |
| Good | 5 | 20.00 |
| General | 11 | 44.00 |
| Poor | 3 | 12.00 |
| Very Poor | 1 | 4.00 |
| **Number of chronic diseases** |  |  |
| 0 | 3 | 12.00 |
| 1-3 | 18 | 72.00 |
| ≥4 | 4 | 16.00 |

^a^yuan: the basic unit of Renminbi (RMB), the currency of China, RMB 1≈US $0.1428.

^b^IC: intrinsic capacity.

^c^Profile 1: multi-subdomain recession-IC imbalance group.

^d^Profile 2: multi-subdomain moderate-sensory deficit group.

^e^Profile 3: multi-subdomain robust-whole balance group
